# Sleep benefits perceptual but not movement-based learning of locomotor sequences

**DOI:** 10.1038/s41598-024-66177-9

**Published:** 2024-07-09

**Authors:** Gabriela Borin, Sumire D. Sato, Rebecca M. C. Spencer, Julia T. Choi

**Affiliations:** 1 Department of Kinesiology, University of Massachusetts Amherst, Amherst, MA USA; 2 Department of Applied Physiology and Kinesiology, University of Florida, PO Box 118205, Gainesville, FL 32611 USA; 3 Neuroscience & Behavior Program, University of Massachusetts Amherst, Amherst, MA USA; 4 Department of Psychological & Brain Sciences, University of Massachusetts Amherst, Amherst, MA USA; 5 Institute for Applied Life Sciences, University of Massachusetts Amherst, Amherst, MA USA

**Keywords:** Gait, Learning, Consolidation, Generalization, Sleep, Human behaviour, Learning and memory, Motor control

## Abstract

Practicing complex locomotor skills, such as those involving a step sequence engages distinct perceptual and motor mechanisms that support the recall of learning under new conditions (i.e., skill transfer). While sleep has been shown to enhance learning of sequences of fine movements (i.e., sleep-dependent consolidation), here we examined whether this benefit extends to learning of a locomotor pattern. Specifically, we tested the perceptual and motor learning of a locomotor sequence following sleep compared to wake. We hypothesized that post-practice sleep would increase locomotor sequence learning in the perceptual, but not in the motor domain. In this study, healthy young adult participants (n = 48; 18–33 years) practiced a step length sequence on a treadmill cued by visual stimuli displayed on a screen during training. Participants were then tested in a perceptual condition (backward walking with the same visual stimuli), or a motor condition (forward walking but with an inverted screen). Skill was assessed immediately, and again after a 12-h delay following overnight sleep or daytime wake (n = 12 for each interval/condition). Off-line learning improved following sleep compared to wake, but only for the perceptual condition. Our results suggest that perceptual and motor sequence learning are processed separately after locomotor training, and further points to a benefit of sleep that is rooted in the perceptual as opposed to the motor aspects of motor learning.

## Introduction

Normal walking is often guided by vision, which provides important information about where to step or not to step^1^. When navigating complex environments (e.g., hiking trail), we must first identify safe footfall locations, and then execute a step sequence to accurately land on successive footfall locations^2^. To accomplish this, the locomotor system must (1) generate rhythmic reciprocal flexor and extensor muscle activity that makes up the basic locomotor pattern, (2) maintain balance by controlling the body center of mass over a changing base of support, and (3) modify each step based on the behavioral goal or context^3,4^. The ability to execute complex locomotor patterns is often disrupted in aging or after neurological damage (e.g., stroke)^5,6^, leading to an increased risk of falls^7^. As such, it is essential to identify ways to enhance locomotion and navigation in those at risk.

In healthy individuals, new locomotor sequences can be acquired through practice, leading to performance gains during training^8–10^. Upper limb studies have shown that motor sequence learning involves the perceptual learning of a sequence of cues and the motor learning of a sequence of movements^11,12^, where a perceptual (or spatial) sequence representation is supported by the parietal-prefrontal cortical loops and a motor (or movement-based) sequence representation is supported by the motor cortical loops^11,13^. Behaviorally, a variant of the serial reaction time task, first introduced by Willingham^12^, has been used to disentangle these parallel contributions to motor sequence learning^12,14^. During training, all subjects responded by pressing the key one position to the left of the stimulus. During testing, the stimulus–response map was changed, so that subjects responded by pressing the key directly corresponding to the location of the stimulus. Transfer to the new condition was demonstrated in one group of subjects using the trained stimulus sequence, but due to the new mapping, subjects would use a different motor response from training. In another group of subjects, the stimulus was shifted to the left of the trained sequence, so that subject would use the same motor response from training. Both groups responded faster in the trained sequence relative to random probes, suggesting that sequence learning is composed of simultaneous perceptual and motor learning^12,14^.

While sequence learning is composed of simultaneous perceptual and motor learning during practice, these components are engaged separately during consolidation^14–17^. Improvements in performance between practice sessions that occur without actively engaging in the task (off-line learning) is thought to reflect consolidation—the process by which a motor memory is enhanced and strengthened after learning^18,19^. Cohen et al. showed that perceptual (also referred to as goal-based) learning improved following overnight sleep (8 p.m. to 8 a.m.), while movement-based learning only improved over wake (8 a.m. to 8 p.m.)^17^. Daytime sleep (nap) also benefited the consolidation of perceptual learning, but not movement-based learning in sequence learning^15,16^. These results are taken to suggest that distinct systems enhance the perceptual and motor learning components, and that sleep plays a crucial role in processing the spatial representation of a sequence. Functional imaging data suggested that the hippocampal and striatal systems support the spatial and motor representations of a motor sequence, respectively^15^. Furthermore, sleep-dependent consolidation of learning and memory has been associated with sleep spindles (EEG bursts prevalent in non-REM sleep)^16,20–23^, including the coupling of sleep spindles with slow oscillations^24,25^, reflecting cortical stabilization of hippocampally-reactivated memories^25^.

Cortical areas involved with sequence learning include the fronto-parietal network that is engaged in the visuospatial mapping of a motor sequence, while bilateral sensorimotor cortices are more involved in the motor representation of sequence learning^11,13,15^. The cortical areas engaged in sequence learning also play an important role in planning and executing adaptive locomotor skills. Specifically, parietal areas have been shown to play an important role in the planning of visually guided step modifications^4,26^. Additionally, activity in the motor cortex may be particularly important for adjusting the timing of muscle activations during voluntary gait modifications^4,27^.

With a few exceptions^28,29^, there is currently a lack of studies focused on sleep-dependent learning in locomotion, which is a fundamental motor behavior. In a study which used an inverted bicycling task to exam sleep’s benefit on gross motor adaptation, it was found that overnight changes in adaptation were greater than performance changes observed following an equivalent interval of wake and were associated with sleep spindles^30^. Here we tested whether such benefits are present in locomotor sequence learning, and whether sleep benefits the perceptual as opposed to the motor aspects of locomotor learning. We hypothesized that sleep will benefit perceptual locomotor sequence learning, as it has been suggested that perceptual learning benefits from hippocampal-neocortical memory stabilization^14^, which is essential for gaining a sleep benefit^31–33^. In contrast, we hypothesized that sleep would not have a significant effect on the motor component of locomotor sequence learning, which is less hippocampal dependent.

## Results

**Figure 1:**
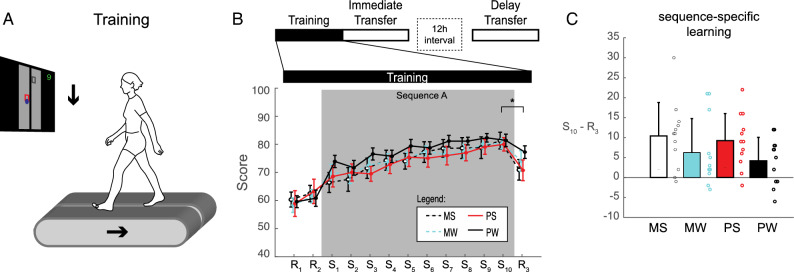
Locomotor sequence learning. (**A**) Participants performed a visually guided walking task that required them to hit virtual targets displayed on the screen by altering step length. The foot position was tracked with retroreflective markers placed on the 5th metatarsal on both feet. The direction of the moving treadmill belts and stepping targets (indicated by the arrows) were congruent during the training period. (**B**) Training consisted of 13 blocks. The first random block (R_1_) was used to familiarize the participant with the visuo-locomotor task. The second random block (R_2_) was used to quantify baseline performance. Participants then practiced Sequence A over 10 blocks (S_1_–S_10_), followed by a last random block (R_3_). Brackets = pairwise comparisons p < 0.05. (**C**) Sequence-specific learning, calculated as the difference in performance (# hits) between S10 and R3 during training, was not different between groups. Error bars are standard deviations.

Participants performed a visually-guided treadmill-walking task that required stepping on virtual targets (Fig. 1A). In this task, participants were required to take a short, medium, or long length step to hit each successive target on the screen. After training in the locomotor sequence task in which the step length followed a 6-item sequence (Sequence A: short-long-medium-long-short-medium), participants were tested (immediately and 12-h later) in one of two new conditions (Fig. 1B). In the perceptual testing condition, the treadmill reversed direction (i.e., backward walking), but the targets remained the same. In the motor testing condition, the screen was inverted upside down, while participants maintained forward walking. The immediate and delayed tests were performed 12-h apart following sleep (e.g., 8 pm to 8am) or wake (e.g., 8am to 8 pm). Young adult participants (n = 48; ages 18–33 years) were assigned to one of four groups: Perceptual-Sleep (PS, n = 12), Perceptual-Wake (PW, n = 12), Motor-Sleep (MS, n = 12), or Motor-Wake (MW, n = 12). Participants’ age, sex, sleep characteristics, and chronotype were not different between groups (Table 1).

**Table 1:** Participant characteristics.

	Motor-sleep (n = 12)	Motor-wake (n = 12)	Perceptual-sleep (n = 12)	Perceptual-wake (n = 12)	p-value
Age	25.3 ± 3.0	21.3 ± 4.0	22.2 ± 3.4	22.8 ± 4.6	0.084
Males:females	6:6	6:6	7:5	5:7	0.881
PSQI	3.2 ± 1.7	4.1 ± 1.3	4.6 ± 1.2	3.8 ± 0.8	0.077
MEQ-SA	52.8 ± 15.9	48.6 ± 11.3	51 ± 7.1	49.6 ± 12.9	0.845
SSS session 1	1.5 ± 0.7	1.8 ± 0.9	1.7 ± 0.8	1.8 ± 0.6	0.823
SSS session 2	1.8 ± 0.7	1.3 ± 0.5	1.8 ± 0.7	1.8 ± 0.6	0.190
Sleep (h)	7.2 ± 0.7	7.4 ± 0.8	6.9 ± 1.1	7.2 ± 0.8	0.628

Mean and standard deviation are reported. *PSQI* Pittsburg Sleep Quality Index (Score range: 0–21; Higher score = worse sleep quality), *MEQ-SA* Morningness-Eveningness Questionnaire Self-Assessment Version (Score range: 16–86; Scores > 58 are “morning types”; Scores < 42 are “evening types”). *SSS* Stanford Sleepiness Scale (Score range: 1–7; Higher score = greater sleepiness). Sleep = Hours of sleep during the night before session 2.

### Locomotor sequence learning

All participants practiced Sequence A over 10 blocks during training, with the treadmill and targets moving in the same (congruent: front of treadmill maps to top of screen) direction (Fig. 1A). Performance (i.e., number of targets hit) on this sequence improved with practice (Fig. 1B). Locomotor sequence-specific learning was indicated by significantly better performance in the last sequence block (S_10_) relative to the last random block (R_3_; Fig. 1B).

Despite being trained at different times of day, sequence-specific learning (S_10_–R_3_) was not significantly different between the four groups of participants (Fig. 1C, F(3,44) = 1.74, p = 0.173). We also confirmed that subjective reports of sleepiness (SSS) were not different between sessions (Session: F(1,44) = 0.03, p = 0.860; Group: F(3,44) = 0.37, p = 0.776; Interaction: F(3,44) = 1.87, p = 0.149) and subjective reports of sleep duration were not different between MS and PS groups (t(22) = 0.67; p = 0.518, d = 0.268).

### Perceptual learning of locomotor sequences

**Figure 2:**
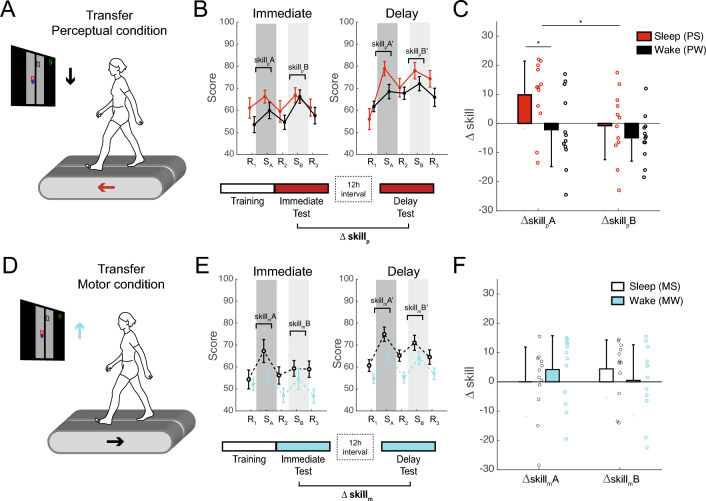
Immediate and Delayed Tests. (**A**–**C**) In the perceptual condition, participants were tested in backward walking (red arrow) with the same visual stimuli. (**D**–**F**) In the motor condition, participants were tested in the same walking direction but reversed visual stimuli (blue arrow). (**B**, **E**). Testing in the perceptual/motor conditions was assessed with Sequence A and Sequence B, immediately and again 12-h later (delayed test) following overnight sleep or daytime wake in the Sleep and Wake groups, respectively. Skill was calculated as #hits for each sequence block relative to the adjacent random blocks, e.g., Skill A = S_A_ – (R_1_ + R_2_)/2. (**C**, **F**) Sleep-dependent learning was determined by comparing offline changes in skill following a 12-h interval with sleep, compared to an equivalent interval awake. e.g., ∆Skill A = Skill A’ (delayed test)—Skill A (immediate test). Subscripts indicate Perceptual or Motor group (i.e., ∆Skill_p_ A = ∆Skill for ‘Sequence A’ for the Perceptual group). *indicate p < 0.05. Error bars are standard deviations. Supplementary Videos show testing in the perceptual and motor conditions.

Skill learning was then tested with the treadmill and targets moving in the opposite (incongruent: front of treadmill maps to bottom of screen) directions. Immediately after training, participants in groups PS and PW were tested in backward walking, while the targets moved in the same direction on the screen as in training (Fig. 2A, Supplementary Video 1). Participants performed better on Sequence A relative to random sequences during backward walking (Skill_p_A), reflecting the perceptual aspect of learning (Fig. 2B). Sequence B (a sequence that was not practiced during training) was also tested in backward walking and showed better performance relative to random sequences (Skill_p_B).

Offline learning was calculated as the difference between immediate and delayed tests. A two-way ANOVA showed that there was greater offline change in perceptual learning (Δskill_p_) for the sleep group compared to the wake group (Fig. 2C, PS vs. PW: F(1,22) = 5.053, p = 0.035), suggesting a benefit of sleep for perceptual learning. There was also greater offline gain for Sequence A compared to the Sequence B (F(1,22) = 5.635, p = 0.027), suggesting that offline gains are dependent on the amount of practice. Pairwise analysis showed a significantly greater Δskill_p_ for the PS group compared to PW for Sequence A (Δskill_p_A: t(22) = 2.401, p = 0.025, d = 0.980), but not for Sequence B (Δskill_p_B: t(22) = 1.016, p = 0.320, d = 0.415). However, the Interval x sequence interaction was not significant (F(1,22) = 1.913, p = 0.181). There was a negative correlation between hours of sleep and Δskill_p_A in Group PS, but this was not significant (p = 0.067, Pearson’s r = − 0.544).

### Movement-based learning of locomotor sequences

Groups MS and MW were tested in walking forward as in training, but the targets moved in a reversed direction on the screen (Fig. 2D, Supplementary Video 2), providing a probe of motor learning. Participants performed better on Sequence A relative to random sequences with an inverted screen (Skill_m_A), reflecting the motor aspect of locomotor sequence learning (Fig. 2E). Sequence B, not practiced during training, also showed better performance relative to random sequences in the motor condition (Skill_m_B).

However for the motor condition, the main effects of Interval type (MS vs. MW: F(1,22) = 0.001, p = 0.970) and sequence type (Sequence A vs. Sequence B: F(1,22) = 0.018, p = 0.895) were not significant nor was the interval x sequence interaction (F(1, 22) = 2.341, p = 0.140) on Δskill_m_ (Fig. 2F).

### Sleep-dependent learning of perceptual but not motor sequence

A two-way ANOVA showed a significant interaction effect (F(1,44) = 5.482, p = 0.024) of Interval (Sleep vs. Wake) on the type on learning (Perceptual vs. Motor). Pairwise analysis showed a significantly greater Δskill_A_ for the PS group compared to PW for Sequence A (Δskill_p_A: t(22) = 2.401, p = 0.025, d = 0.980), but not for the MS group compared to MW for Sequence A (Δskill_m_A: t(22) = − 0.880, p = 0.388, d = − 0.359).

## Discussion

The objective of this study was to examine the role of sleep on locomotor learning in healthy young adults. Specifically, we used a novel variant of a paradigm that dissociates perceptual and motor learning of locomotor sequences^9^. We found that sleep benefited perceptual but not motor-based learning. Our results demonstrate that locomotor sequence learning, like upper limb (arm, hand) sequence learning, seems to comprise learning across two dimensions: learning the sequence of movement responses (motor or muscle-based learning) and learning the sequence of response goals (perceptual or goal-based learning)^12^. These findings suggest that there are distinct neural systems that support improvement in performance in the motor and perceptual domains, reflected in different offline gains for motor and perceptual skills over 12-h intervals.

### Sequence learning of complex lower-body movement

Compared to isolated upper limb tasks often used to investigate motor sequence learning, whole-body movements pose additional task demands such as interlimb coordination and postural control. Previous studies have adapted the serial reaction time task to be performed by stepping on floor targets in a sequential pattern^34,35^, which requires continuous changes in postural dynamics. It was found that increases in resting-state functional connectivity between the lateral prefrontal cortex and associative striatum were correlated with sequence-specific improvements during the acquisition of complex lower-body movements^34^. Furthermore, motor imagery practice (without physical practice) can improve performance in the sequential footstep task^35^, reflecting goal-based learning. These studies have thus far suggested that perceptual and motor mechanisms support learning across simple single-body part and more complex lower-body motor tasks.

Sequence-specific learning can be integrated with walking^9^, which has a built-in rhythmicity not characteristic of other voluntary movements. During walking, perceptual and motor learning processes must interact with a hierarchy of locomotor centers that are responsible for maintaining continuous reciprocal gait, modifying steps to changing behavioral goals, and regulating postural control during walking. While the basic reciprocal locomotor rhythm and pattern is coordinated by spinal pattern generators and brainstem locomotor centers^36,37^, the precise timing and amplitude of muscle activations during goal-directed walking is regulated by the posterior parietal cortex that plans abstract task-level goals, and the motor cortex that determine how step modifications are executed^4^.

The current experiments showed that participants maintained better performance on a practiced sequence relative to random sequences when switching to backward walking. The transfer of locomotor sequence-specific learning to backward walking strongly suggests that perceptual mechanisms are involved during the recall of previously learned visual sequences, given that our previous findings showed independent neural circuits for forward and backward walking adaptation in humans^38^. The current experiments also unraveled the motor aspect of locomotor sequence learning, which enabled participants to maintain performance on the practiced sequence when the visual cues were inverted upside down. These results demonstrate that there are separate perceptual and motor mechanisms underlying locomotor sequence learning.

Practicing Sequence A also facilitated the subsequent perceptual and motor learning of a different Sequence B in walking. Sequence B, not practiced during training, showed better performance relative to random sequences immediately after training. The practiced Sequence A and the new Sequence B both consisted of short, medium, and long step lengths, and the sequence repeated every 6 steps. In this case, practicing Sequence A might have facilitated Sequence B due to similar sub-patterns (e.g., S-L-M) embedded in both step length sequences.

### Sleep-dependent perceptual learning of locomotor sequences

The consolidation of perceptual learning is thought to be sleep-dependent^14,16,17^. Our results indeed found that off-line improvements between immediate and delayed tests were greater following sleep compared to wake for perceptual-based locomotor sequence learning. Sleep-dependent consolidation of perceptual learning has also been demonstrated following motor imagery practice of a sequential footstep, whereas physical practice did not improve performance after a night of sleep^39^. These behavioral studies suggests that perceptual learning of lower-body skills does benefit from overnight sleep.

Off-line perceptual learning was greater for Sequence A compared to the new Sequence B, which was only performed briefly before a 12-h break. If performing a new sequence after the training sequence caused retrograde interference, the off-line gain for Sequence A may have been eliminated. However, this was not the case. To rule out the lack of interference we would need to test a separate control group (subjects who only perform Sequence A). Nonetheless, the current results suggest that the perceptual learning of a practiced sequence benefits from sleep.

In this study, we did not find a significant correlation between hours of sleep following practice and offline gains in perceptual learning. However, sleep duration was quantified by self-report only. One limitation of the current study is that we did not obtain polysomnography during the sleep intervals. Changes in procedural memory following sleep have been associated with non-REM stage 2 (nREM2) sleep and sleep spindles (EEG bursts prevalent in nREM2)^20,40^. More recent studies on gross motor skill learning have found a correlation between improved performance accuracy with increased nREM2 sleep spindles^30^, this echoes sleep mechanisms that may support consolidation of perceptual learning across fine motor and whole-body motor skills. Future studies including measures of sleep architecture and microstructure are essential to understanding the specific mechanism underlying this sleep-related improvement for locomotor learning.

### Offline movement-based learning of locomotor sequences

Off-line improvements between immediate and delayed tests for movement-based locomotor sequence learning were not significantly different between sleep and wake. This finding is consistent with our hypothesis that the motor component would not benefit from sleep. On the other hand, it has been suggested that consolidation of movement-based learning is wake-dependent^14,17^. However, our results found evidence for neither sleep- nor wake-dependent improvements of movement-based locomotor sequence learning. Our results suggest that movement-based learning of locomotor sequences is stable off-line. One potential explanation for the lack of wake-dependent consolidation in this study could be that wake consolidation primarily improves speed rather than accuracy^11^. It is important to note that the offline gains reported were based on accuracy, not speed (since cadence was dictated by the prescribed step length and treadmill speed). A locomotor sequence task that does not constraint cadence (e.g., overground walking) could be used to obtain performance measures reflecting speed improvements, in order to test this prediction.

### Neural mechanisms for locomotor sequence learning

Understanding the neural mechanisms underlying perceptual and motor learning of locomotor sequences is important for advancing gait interventions. Central pattern generators maintain the basic rhythmic pattern of walking, but their activity can be modulated by supraspinal inputs to incorporate learned sequences^3,9,36^. Human functional imaging studies showed that cortical contributions to sequence learning involve the posterior parietal cortex, primary motor cortex, supplementary motor area, and frontal regions^11,41,42^. Learning is optimized by the basal ganglia and the cerebellum, where signals from the cerebral cortex are processed in terms of their reward value and sensorimotor accuracy^11^.

Sequence learning may initially involve a circuit comprising the association (prefrontal and parietal) cortices and the basal ganglia which acquires that spatial sequence quickly; whereas the motor sequence is acquired more slowly and involves the motor cortices (M1, SMA) and basal ganglia^11^. During walking, the posterior parietal cortex plays an important role in spatial planning, in a limb-independent manner, ensuring that movements are accurately adapted to the environment^26,43,44^. This aligns with its role in perceptual learning. The motor cortex regulates the duration, level, and timing of small groups of synergistic muscles, active at different times during the gait modification^4,27,45^. The precise control of muscle activation by the motor cortex is essential for smooth, coordinated complex walking patterns, which aligns with the motor component of sequence learning.

## Conclusion

Previous studies of sequence learning have mostly focused on upper-limb motor tasks. Our study provides a new perspective by investigating locomotor sequence learning. It demonstrates that distinct neural mechanisms underpin motor and perceptual learning of locomotor sequences. The results provided new insights into the role of sleep in the consolidation of learning involving the lower limbs. We posit that incorporating sleep after gait rehabilitation may enhance the transfer of perceptual learning to novel environments. The findings have practical implications for refining neurorehabilitation strategies and enhancing recovery of patients with walking difficulties.

## Methods

### Participants

Forty-eight healthy young adults (18–33 years) with no known neurological disorders participated in this study (Table 1). Participants were screened for visual acuity (must be over 20/40 to participate) using the Snellen eye chart. At the beginning of each session, participants completed the following assessments to evaluate their sleep characteristics: Stanford Sleepiness Scale (SSS) for a subjective measure of their sleepiness at the beginning of each experimental session^46^, the Morningness-Eveningness Self-Assessment Questionnaire (MEQ-SA) to determine chronotype related to their circadian rhythms^47^, and Pittsburg Sleep Quality Index (PSQI) to assess their sleep quality in the past month^48^. Participants also reported the approximate number of hours they slept the night before each session. All participants gave informed written consent before the study in accordance with the protocol approved by the Institutional Review Board of University of Massachusetts Amherst (Protocol # 2018-4513) and according to the Declaration of Helsinki.

### Locomotor task

The task utilized an instrumented split-belt treadmill (Bertec, Columbus, OH). A projector screen (50″ × 50″) placed 150 cm in front of the treadmill displayed virtual stepping targets and a cursor representing the current position of the foot in swing. A custom-made program controlled the distance between successive targets (i.e., step length) and retrieved real-time motion-capture data from the QTM server (Qualisys, Göteborg, Sweden), which streamed marker position and ground reaction force data while subjects walked. Based on our previous studies^9,49^, the treadmill speed for each participant was normalized to their leg length to match cadence (100 steps/minute) across subjects. Stepping targets for the left foot and right foot, represented by 16 cm × 16 cm open squares, appeared on the left and right of midline, respectively. Both the target for the current leg in swing phase (red square) and the subsequent target for the contralateral limb (grey square) were shown on the screen. The screen displayed an 8 cm diameter circle representing the y-position (anterior–posterior) of the swing foot (i.e., marker on the fifth metatarsal).

Participants were instructed to step on the virtual targets as accurately as possible, by adjusting their step length. A successful hit was one where the center of the foot (circle) was within 6 cm of the center of the target (square) after heel-strike. Participants received a point each time the foot successfully hits the target. The updated score was displayed on the top right corner of the screen.

### Sequence learning blocks

Locomotor sequences were comprised of three different step lengths: short (S), medium (M), and long (L). We used two sequences: Sequence A (S_A_) : S–L–M–L–S–M: and Sequence B (S_B_): S–M–L–S–L–M. The medium step length was set at 2/3 leg length, measured from the greater trochanter (hip) to the medial malleolus (ankle) and averaged between limbs. The short step length was 80% and the long step length was 120% the medium step length. Each block consisted of 100 steps, where subjects were presented with targets that required either a random or repeating sequence of step lengths. Participants performed 10 blocks with the same sequence (S_A_) during training. During testing, participants performed the same sequence (S_A_) and a new sequence (S_B_) under the perceptual or motor condition. Random blocks (R) were used to probe general performance (sequence independent learning) during training and testing.

### Experimental groups

All participants completed two sessions, separated by 12 ± 2 h. Participants were randomly assigned to one of four groups: Motor-Sleep (MS), Motor-Wake (MW), Perceptual-Sleep (PS), or Perceptual-Wake (PW). For the Wake groups (MW and PW), training and immediate test during the first session took place in the morning, and the delayed test during the second session occurred on the same day in the evening, following daytime wake. For the Sleep groups (MS and PS), training and immediate test took place in the evening and the delayed test occurred the next day in the morning, following overnight sleep. This allowed us to compare changes in skill transfer over an equivalent period with and without sleep.

Immediate and delayed post-tests were performed under the perceptual or motor conditions. To probe perceptual learning, participants in groups PS and PW were tested walking backward (treadmill moving forward) with the targets going in the same direction on the screen as in training (i.e., the visual stimulus was the same, but the movement required was different)**.** To probe motor learning, participants in groups MS and MW were tested walking forward as in training, but the targets moved in an opposite direction on the screen (i.e., the visual stimulus was changed, but the same leg movement was required)**.**

### Data analysis

Sequence-specific learning during training was calculated as the difference in performance (number of successful hits) between the last training block (S_10_) and the last random block (R_3_). Skill transfer was calculated as Skill A = S_A_ − (R_1_ + R_2_)/2 and Skill B = S_B_ − (R_2_ + R_3_)/2, during the Immediate and Delayed tests. A difference index (ΔSkill = Delay − Immediate) was calculated for Skill_p_ A, Skill_p_ B, Skill_m_ A, and Skill_m_ B. Subscripts indicate Perceptual (i.e., Skill_p_ A/B) or Motor (i.e., Skill_m_ A/B) groups.

A one-way ANOVA was used to test group differences in sequence-specific learning (S_10_–R_3_) during Training. Two-way mixed-measures ANOVAs were used to test the main effect of Interval (Sleep vs. Wake) and test sequence (S_A_ vs. S_B_), and their interaction effect on ΔSkill for the Motor and Sleep groups, separately. Correlation between hours of sleep and ΔSkill_p_A (ΔSkill for Sequence A for the Perceptual group) was assessed for the Perceptual-Sleep group with a Pearson’s Correlation test. We also tested for differences in the effects of Interval (Sleep vs. Wake) on the two types of learning (Perceptual vs. Motor) using a two-way ANOVA.

To assess sex distribution difference between groups, a chi-squared test of independence was used. An independent samples t-test was used to assess sleep duration differences between MS and PS groups. One-way ANOVAs were used to analyze between-group differences in age, PSQI, MEQ-SA, and SSS. Additionally, a repeated-measures ANOVA was used to assess possible differences in the SSS between two sessions. All statistical analyses were performed on JASP software (Version 0.14.1; University of Amsterdam). α value of 0.05 was used for all statistical analyses.

### Power estimation

A priori power analysis was conducted with G*Power software (version 3.1.9.6, Dusseldorf, Germany) to determine the appropriate sample size for this study^50^. We used the means and standard deviations from Albouy et al.^16^ to calculate effect size for the difference in means between sleep and wake groups for perceptual learning (d = 1.388). The analysis showed that n = 12 per group can achieve a power > 0.9 to detect differences between sleep vs. wake groups for perceptual learning.

### Supplementary Information

Supplementary Information.

## Data Availability

The dataset from the current study are available from the corresponding author on reasonable request.
