# Supplementary material for: Sleep benefits perceptual but not movement-based learning of locomotor sequences
Source: Sci Rep. 2024 Jul 9;14:15868. doi: 10.1038/s41598-024-66177-9 (PMC11233676; doi:10.1038/s41598-024-66177-9)
Supplement: Supplementary file 1 — Supplementary Information. [file 41598_2024_66177_MOESM1_ESM.docx]

**Supplemental Material**

Supplemental Video 1: <https://doi.org/10.6084/m9.figshare.25148402.v1>

Supplemental Video 2: <https://doi.org/10.6084/m9.figshare.25148411.v1>
